# Detection of inflammation-related blood–brain barrier dysfunction using PET and MR imaging: a pilot study

**DOI:** 10.1038/s41598-026-47352-6

**Published:** 2026-04-10

**Authors:** Colmar F. Hilbrig, Bernd Baumann, Wolfgang Sievert, Rebecca Halbgebauer, Markus Huber-Lang, Ambros J. Beer, Volker Rasche, Jessica Löffler

**Affiliations:** 1 Department of Nuclear Medicine, University Hospital Ulm, Albert-Einstein Allee 23, 89081 Ulm, Germany; 2 Institute of Physiological Chemistry, Ulm University, Ulm, Germany; 3 Institute for Clinical and Experimental Traumatology, Ulm University Hospital, Ulm, Germany; 4 Center for Translational Imaging, Core Facility Small Animal Imaging, Ulm University Hospital, Ulm, Germany; 5 Department of Internal Medicine II, Ulm University Hospital, Ulm, Germany

**Keywords:** BBB-dysfunction, Neuroinflammation, Albumin, PET/MR imaging, Biological techniques, Biomarkers, Diseases, Medical research, Neurology, Neuroscience

## Abstract

The purpose of this study is to non-invasively image subtle blood–brain barrier (BBB) permeability changes in a state of chronic neuroinflammation using radiolabelled human serum albumin (HSA). Although biomarker-based methods for detecting BBB dysfunctions have been established, sensitive spatial and quantitative imaging of low-grade, chronic BBB permeability changes remains limited. This study seeks to establish radiolabelled albumin as a sensitive radiotracer for visualizing and quantifying BBB dysfunction during chronic neuroinflammation. This study evaluated the utility of radiolabeled serum albumin ([^89^Zr]Zr-DFO-HSA) for assessing BBB permeability using co-registered PET and MR imaging in a transgenic mouse model of neuroinflammation mediated by IKK/NF-κB activation. PET and MRI revealed significantly increased radiotracer accumulation in the cerebellum of neuroinflammatory mice, corroborated by *ex vivo* analyses. Quantitative PET data revealed elevated intracerebral radiotracer concentrations over 24 h, even in the absence of morphological changes in histological evaluations. These findings emphasize the sensitivity of [^89^Zr]Zr-DFO-HSA in detecting early-stage BBB permeability changes prior to the onset of overt morphological damage or clinical symptoms. As a surrogate marker for macromolecular leakage, this non invasive imaging approach shows promise for advancing preclinical research into neuroinflammatory disorders and barrier dysfunctions.

**Supplementary Information:**

The online version contains supplementary material available at 10.1038/s41598-026-47352-6.

## Introduction

Neuroinflammation is an inflammatory reaction of the central nervous system (CNS), driven by brain-resident and peripheral immune cells responding to various triggers including acute trauma, infection, and neurodegenerative diseases^1^. Neuroinflammation is primarily initiated by the activation of glial cells, *e.g.* microglia and astrocytes, which release pro-inflammatory mediators like cytokines and reactive oxygen species (ROS)^2^. While acute neuroinflammation can be protective by reducing neuronal injury, persistent or chronic inflammation often exhibit harmful consequences, leading to synaptic dysfunction and neuronal death^3,4^. The blood–brain barrier (BBB) plays a key role in this dynamic, as inflammation-induced BBB disruption promotes peripheral immune cell infiltration, thereby perpetuating and exacerbating the current inflammatory state of the CNS^5,6^. Recent advances in understanding glial cell activation, BBB dysfunction, and synaptic damage emphasizes the complex and ambivalent role of neuroinflammation.

The clinical significance of addressing neuroinflammation and BBB integrity is evident in a wide range of neurological and psychiatric disorders, as both processes critically influence disease progression and treatment outcomes^7,8^. Exemplified in neurodegenerative diseases, neuroinflammation is both an underlying cause and consequence of neuronal damage and cognitive decline. Similarly, in traumatic brain injury, neuroinflammation and BBB disruption exacerbate secondary injury, impacting recovery and long-term prognosis^9,10^. Detecting and characterizing neuroinflammations, specifically focusing on the complex interplay between inflammatory processes and BBB integrity have been attempted by several innovative approaches. For instance, components of the extracellular matrix have been investigated as potential blood-based biomarkers of BBB structural compromise during neuroinflammation^11–13,14^. Similarly, pro-inflammatory cytokine profiles have been increasingly associated with altered vascular permeability in neurodegenerative conditions such as Alzheimer’s disease. These observations are further supported by the detection of elevated human serum albumin (HSA) in the cerebrospinal fluid, which serves as a clinical hallmark of the link between neuroinflammation and increased protein extravasation into the CNS^15–17^. While these blood-based biomarkers represent a significant step forward, further refinement and validation are necessary for clinical use, specifically considering the inherent limitations in accurate quantification and precise localization of BBB leakage. Direct approaches for detecting neuroinflammation have significantly advanced, particularly in the field of multimodal PET imaging, as this imaging modality offers high sensitivity and robust quantitative capabilities^18,33^ consequently, radiotracers have been developed and extensively studied as diagnostic tools to specifically target neuroinflammatory processes. Although these radiotracers facilitate the detection of glial activation and the overall neuroinflammatory state, their inherently high lipophilicity allows them to readily cross the BBB via passive diffusion, thereby limiting their ability to specifically evaluate and quantify BBB leakage or integrity^19,37^.

In this study, we explore the use of radiolabeled HSA ([^89^Zr]Zr-DFO-HSA) as a diagnostic radiotracer to investigate the early-stage effects of chronic neuroinflammation on BBB integrity in a mouse model, utilizing multimodal PET and MR imaging.

## Materials and methods

### Radiosynthesis and radiolabeling

The radiosynthesis and radiolabeling followed the protocol by Hilbrig et al*.*^21^. The radioactive isotope [^89^Zr]Zr^4+^, obtained as zirconium-89 oxalate ([^89^Zr]Zr(C_2_O_4_)_2_) from PerkinElmer Inc. (Waltham, MA, USA), was purified using an anion exchange cartridge (Sep-Pak Accell Plus QMA Plus Light, Waters GmbH, Eschborn, Germany) and eluted as [^89^Zr]ZrCl_4_, to remove the toxic effects of oxalate^22^. Subsequently, [^89^Zr]ZrCl_4_ was dissolved in 1 M HCl (Merck KGaA, Darmstadt, Germany) and adjusted to a pH 6–7 using 1 M Na_2_CO_3_ solution (Merck KGaA, Darmstadt, Germany). For stable complexation of zirconium-89 with human serum albumin (HSA), the chelating agent desferal (Sigma-Aldrich, St. Louis, Missouri, USA) was used. The covalent attachment of desferal (DFO) to HSA followed Vosjan et al.^26^ with slight modifications and is detailed in the Supplements (S.1).

### Assessment of the effects of chronic neuroinflammation on the integrity of blood–brain-barrier using PET-MRI

#### Animal studies

Blood–brain-barrier (BBB) studies were performed in male NMRI transgenic mice of the GFAP.tTA x (tetO)7.IKK2-CA (IKK2-CA^GFAP^) strain (n = 3; mean weight (39.7 ± 1.6) g) and male NMRI controls (n = 4; mean body weight (38.9 ± 4.2) g). One transgenic animal was excluded from the analysis due to misinjection. Experiments were conducted on 16-week-old mice. In the transgenic group, neuroinflammation had been chronically induced for 12 weeks prior to assessment. All animal experiments were conducted in accordance with institutional and national regulations and are reported in compliance with the ARRIVE guidelines. Measures were implemented throughout the study to minimize animal discomfort and distress. Mice were housed under standard laboratory conditions in species-appropriate environments, with provision of nesting materials, refuges, and environmental enrichment. Experimental procedures were designed to be minimally invasive, and animals were monitored regularly for signs of pain, stress, or distress. Predefined humane endpoints were established prior to the study and applied when necessary to ensure animal welfare. All procedures were carried out by trained and qualified personnel.

Global neuroinflammation was established by postnatal NF-κB activation in astrocytes^23,35^. The proinflammatory IKK2/NF-κB signaling pathway was activated utilizing the tetracycline-regulated transgene system (tet-off system) at the age of 5 weeks by doxycycline removal. Concurrently, luciferase was expressed, allowing the neuroinflammatory cascade to be monitored via the bioluminescence signal (data not shown)^4^. Prior to the in vivo PET-MRI analysis of [89Zr]Zr-DFO-HSA, mice were anesthetized in a 2.5% isoflurane and compressed air/oxygen (80%/20%) flooded mouse anesthesia chamber. After confirmation of an adequate depth of anesthesia by the absence of the set-up reflexes^36^, mice were maintained under anesthesia with 2% isoflurane via mask.

Subsequently, 150 µL [^89^Zr]Zr-DFO-HSA (0.50 ± 0.07 µg/mL, 1.33 ± 0.35 MBq; median: 1.35 MBq) was injected intravenously via a catheter (30 G) in the lateral tail vein, allowing real-time biodistribution monitoring. The dosage was specifically chosen to balance high-quality PET imaging with the 3R principle, thereby minimizing the cumulative radiation burden and associated physiological distress. To ensure optimal image quality and precise quantification, a 60-min acquisition protocol was performed. After the initial dynamic PET-scan was completed (60 min p.i.) the mouse, catheter, and syringe were separately measured using a dose calibrator to determine the applied radioactivity (100% injected activity [%IA]). Additionally, for anatomical reference, mice were examined using MRI. Both measurements were repeated 24 h *p.i.* using the same protocol. No additional tracer injection was required due to the long physical half-life of zirconium-89. The in vivo stability of the [^89^Zr]Zr-DFO-HSA construct was previously validated in mice by Hilbrig et al*.*^21^, ensuring that the delayed scans accurately reflect the distribution of the intact radiotracer rather than free zirconium-89.

#### In vivo dynamic PET imaging and image reconstruction

Dynamic PET scans were performed to evaluate the in vivo biodistribution of [^89^Zr]Zr-DFO-HSA via small animal PET scanner Focus120. The scanner provides a high spatial resolution (< 1.3 mm) and high sensitivity (approximately 7%) with a 12 cm diameter bore and axial field of view length of 7.6 cm^31^. PET data were acquired in list-mode format and subsequently organized into 3D sinograms for reconstruction. For the initial 60-min dynamic scan, 23 time frames were defined (6 × 20 s, 7 × 60 s, and 10 × 300 s). For the late-phase scan at 24 h *p.i.,* data were reconstructed into 12 frames of 5 min each. Reconstructions were performed with OSEM3D/MAP using 4 OSEM2D, 2 OSEM3D, and 18 MAP iterations with a matrix of 256 × 256 and a zoom factor of 1.5, resulting in a voxel spacing of 0.39 × 0.39 × 0.8 mm^3^.

#### Anatomical and high resolution MR imaging analysis

Morphological assessments were performed via high resolution MRI. MRI measurements on an 11.7 T small-animal system (Bruker BioSpec 117/16, Bruker BioSpin, Ettlingen, Germany). Whole-body anatomical imaging was conducted with a 60 mm transmit/receive (T/R) volume resonator using a multislice FLASH sequence with the following parameters: repetition time (TR) = 150 ms, echo time (TE) = 1.5 ms, flip angle (FA) = 15°, matrix size = 750 × 300, in-plane resolution = 100 × 133 µm^2^, slice thickness = 500 µm, and 12 signal averages (NSA). A total of 40 coronal slices were acquired. For high-resolution cranial imaging, a 4-element phased-array mouse brain coil was used in combination with a 72 mm volume resonator. The acquisition parameters were: TR = 150 ms, TE = 1.5 ms, in-plane resolution = 65 µm, slice thickness = 0.5 mm, and 8 signal averages (NSA). All images were assessed for parenchymal abnormalities and structural integrity.

#### Quantification of PET data

Volumes of interest (VOIs) for the heart, brain, blood, and whole body were manually defined based on co-registered PET and MR images using PMOD software (PMOD Technologies, Zurich, Switzerland). All radioactivity data were decay-corrected to the time of injection. To facilitate direct comparison with ex vivo biodistribution data, PET radioactivity concentrations were expressed as a percentage of the injected activity per milliliter (%IA/mL) and decay-corrected to the time of injection. Time-activity curves (TACs) were subsequently generated from the dynamic PET data to characterize the radiotracer kinetics over the total measurement period.

To evaluate the intracerebral accumulation of the radiotracer relative to systemic circulation, brain-to-blood activity concentration ratios were calculated at 1 h and 24 h *p.i.*. These ratios were expressed as mean ± standard deviation (SD). Comparative analysis was performed to characterize the differential tracer distribution between the neuroinflammatory and control cohorts at these specific time points.

#### *Ex vivo* validation and quantification of radiotracer accumulation within murine organs

Following in vivo PET imaging, the mice were euthanized, and brains, blood samples, and solid organs were harvested. The excised brains, along the heart, lungs, kidneys, spleen, colon, small intestine, muscle, bone, and tail, were washed in PBS to reduce residual blood radioactivity. Organ weights were recorded [g]. Radioactivity was quantified through γ-counter analysis (COBRA II γ-counter (PerkinElmer Inc., Waltham, MA, USA)), decay-corrected to injection time, and normalized to total injected activity (%IA) and organ weight (%IA/g). Results were given as mean ± SD and median and presented in dot plots, allowing for the inspection of individual animal values.

Hematoxylin and eosin (H&E) staining and digital autoradiography (DAR) were performed to assess intracerebral morphology and visualize cerebral zirconium-89 distribution; the general protocol is outlined in accordance to Hilbrig et al.^21^and is detailed in the Supplements (S.1.).

### Statistical evaluations

Statistical analyses were conducted using GraphPad Prism (Ver. 9.4.1, GraphPad Software, San Diego, CA, USA). Given the exploratory, proof-of-concept nature of this study and the limited cohort size (n = 3 neuroinflammatory group; n = 4 control group) the Mann–Whitney U test was employed to provide preliminary indications of statistical differences. Results are expressed as mean ± standard deviation (SD). To ensure transparency and allow for a detailed assessment of data variability, individual animal data points are provided in the supplementary material (S.2 table 1–8). Statistical significance was defined as *p* < 0.05.

## Results

### In vivo biodistribution analysis of [^89^Zr]Zr-DFO-HSA within the mouse model

**Figure 1:**
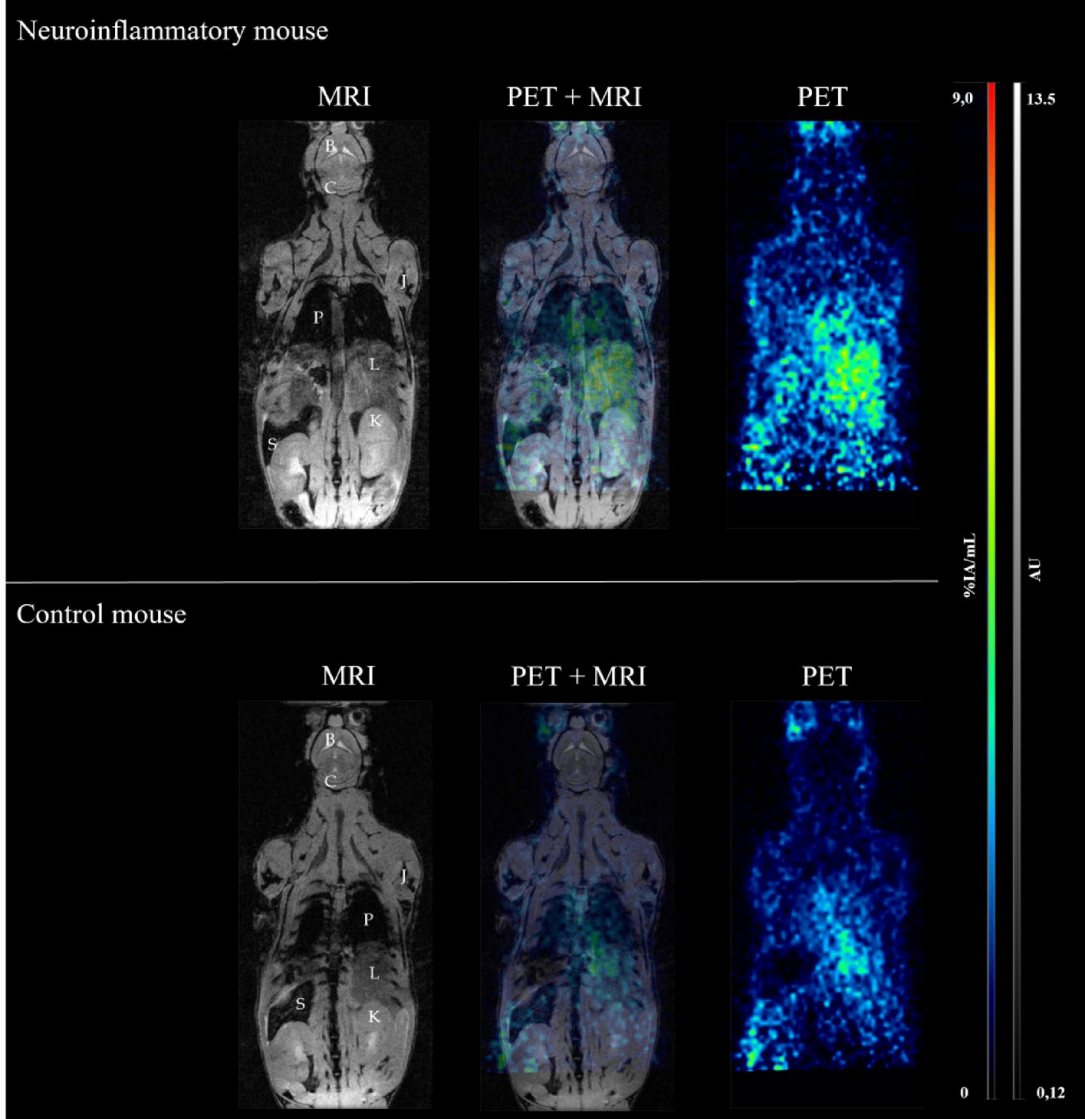
PET and MRI analysis of neuroinflammatory and control mice. MR imaging (left column), PET imaging (right column) and fused PET/MR imaging (middle column) at 24 h p.i. of an exemplary neuroinflammatory mouse (top row) and a control mouse (bottom row). MR imaging revealed no apparent morphological differences in the cerebellum (C), brain (B), liver (L), spleen (S), or kidneys (K) between groups. PET imaging demonstrated comparable radiotracer biodistribution in neuroinflammatory and control mice, with strong radiotracer uptake in abdominal organs, particularly the liver, in both groups. Cerebral signal was weak in the neuroinflammatory mouse and negligible in the control mouse. No radiotracer accumulation was observed in the joints (J) in either model.

Anatomical full-body MR imaging (Fig. 1, left column) revealed no morphometric differences between the two mouse models, with all organs visually exhibiting comparable organ volumes and parenchymal signal intensity.

**Figure 2:**
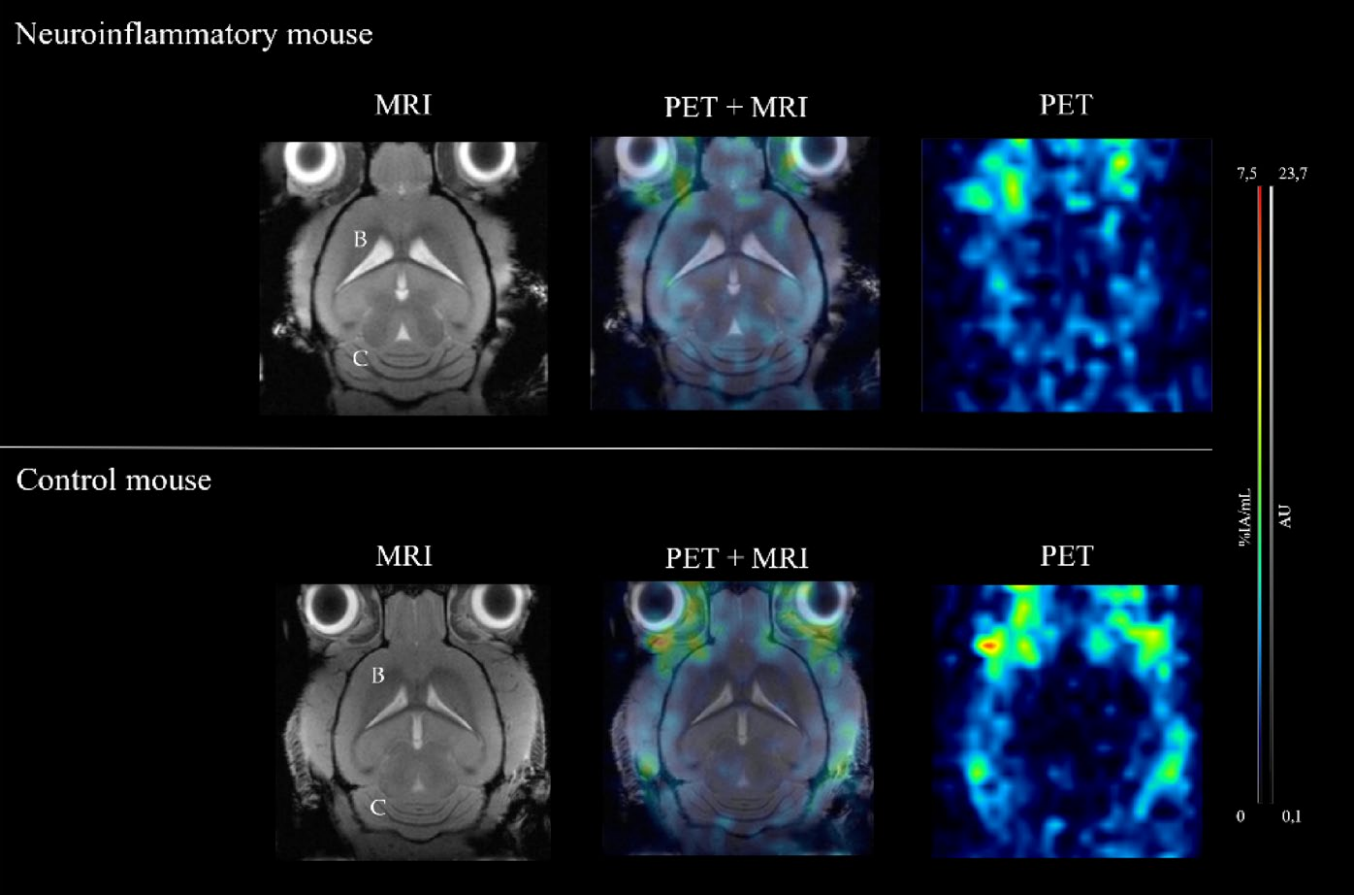
Cranial PET and MRI analysis of neuroinflammatory and control mice. MR imaging (left column), PET imaging (right column), and fused PET/MR overlay images (middle column) at 24 h *p.i.* of an exemplary neuroinflammatory mouse (top row) and a control mouse (bottom row). No morphological differences were observed in the brain (B) or cerebellum (C). PET imaging depicted subtle cerebellar radiotracer signal in the neuroinflammatory mouse, which was absent in the control.

Specifically, despite chronic neuroinflammation being expected to manifest most prominently in the brain^8,26^, detailed cranial MRI showed no detectable abnormalities or parenchymal changes (Fig. 2, left column). The biodistribution of [^89^Zr]Zr-DFO-HSA appeared similar across both models, characterized by high radiotracer accumulation in well-perfused abdominal organs, particularly the liver (Fig. 1, right column). While detailed cranial PET imaging of control mice revealed negligible intracerebral signal with no regional enhancement, the neuroinflammatory mice exhibited a distinct radiotracer pattern localized to the cerebellum (Fig. 2, right column).

**Figure 3:**
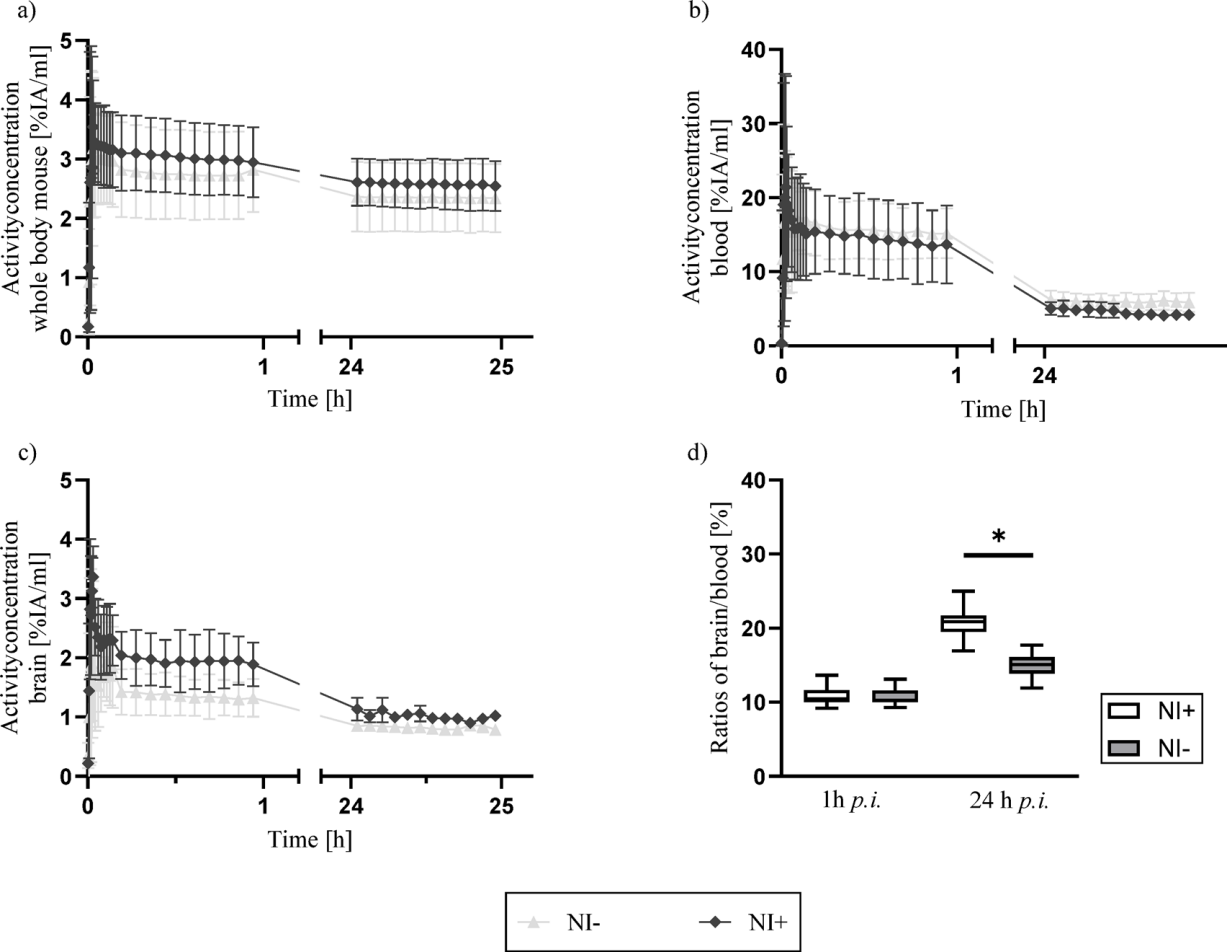
In vivo biodistribution of radiotracer over 24 h in neuroinflammatory (NI +) and control mice (NI -). Time–activity curves (TACs) for (**a**) total body and (**b**) blood, showing a comparable decline in radiotracer concentration in both groups. (**c**) Brain TACs indicated that intracerebral tracer concentrations also declined over time in both groups but remained higher in neuroinflammatory mice at 24 h p.i.. (**d**) Brain-to-blood ratios were similar at early time points (1 h p.i.) and became significantly higher in the neuroinflammatory model at 24 h p.i.. Statistically significant differences are indicated by ⋆.

The corresponding time-activity curves (TACs) indicated similar radiotracer kinetic profiles across both groups (Fig. 3). In control mice, the mean whole-body activity concentration was (4.05 ± 0.26) %IA/mL at 60 min *p.i.*, declining to (3.00 ± 0.05) %IA/mL by 24 h *p.i.* (*p* < 0.001; Fig. 3a). Similarly, the neuroinflammatory model exhibited an initial mean activity concentration of (4.36 ± 0.76) %IA/mL at 1 h *p.i*., which decreased to (3.02 ± 0.15) %IA/mL at 24 h *p.i.* (*p* < 0.001). No significant differences in whole-body clearance dynamics were observed between the two models (*p* = 0.95).

Blood time-activity curves (TAC; Fig. 3b) for the neuroinflammatory group peaked immediately post-injection ((27.43 ± 1.75) %IA/mL) and decreased continuously at a rate of -0.56 (%IA/mL)/h (R^2^ = 0.96), reaching (7.62 ± 0.76) %IA/mL by 24 h *p.i.*. The control group exhibited comparable pharmacokinetics, with an initial concentration of (22.07 ± 0.63) %IA/mL and a decline rate of -0.41 (%IA/mL)/h (R^2^ = 0.96), resulting in (6.94 ± 0.56) %IA/mL at 24 h *p.i.*.

The brain TAC (Fig. 3c) of the neuroinflammatory model suggested an initial activity concentration of (2.01 ± 0.07) %IA/mL at 1 h *p.i*., decreasing by -0.04 (%IA/mL)/h (R^2^ = 0.81) to (1.24 ± 0.11) %IA/mL at 24 h *p.i*. (*p* < 0.001). In contrast, controls model exhibited an initial intracerebral tracer accumulation of (1.60 ± 0.06) %IA/mL, which decreased to (0.88 ± 0.04) %IA/mL (*p* < 0.001) after 24 h *p.i*., with an average reduction rate of -0.04 (%IA/mL)/h (R^2^ = 0.70). Comparative analysis indicated significantly higher intracerebral tracer concentrations in the neuroinflammatory mice compared to the control model (*p* < 0.001). Brain-to-blood ratios were calculated to further evaluate intracerebral distribution. Initially, both models demonstrated comparable ratios (neuroinflammatory: (10.32 ± 0.65) %; control: (10.27 ± 0.63) %). However, by 24 h *p.i.,* the ratio in the neuroinflammatory model increased to (17.46 ± 1.62) %, while the control group reached (13.12 ± 1.42) %. Comparative analysis confirmed that the brain-to-blood ratios were significantly higher in the neuroinflammatory mice compared to controls (*p* < 0.001).

### *Ex vivo* validation

**Figure 4:**
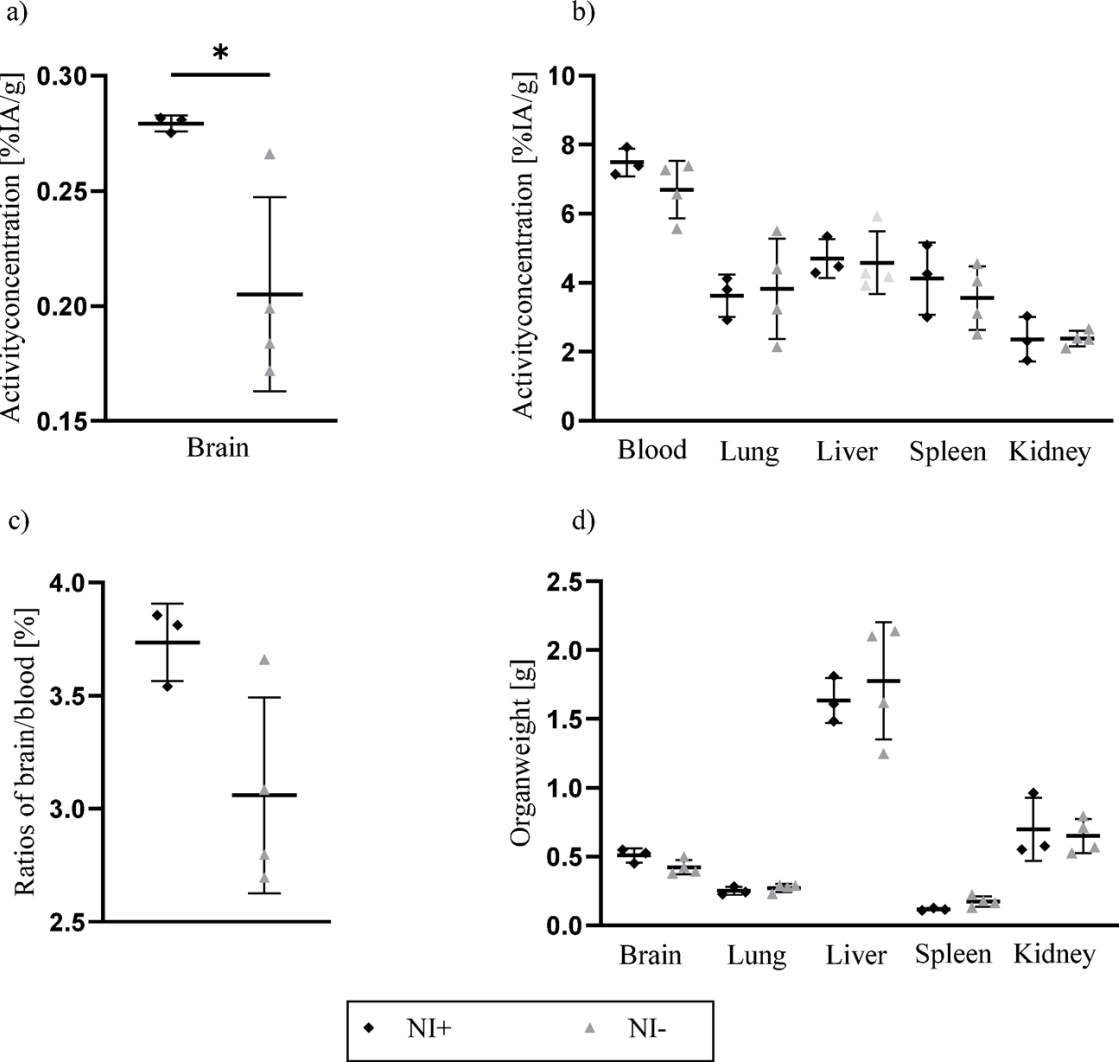
Ex vivo biodistribution analysis 24 h *p.i.* in neuroinflammatory (NI +) and control mice (NI-). (**a**) Brain uptake revealing higher intracerebral activity concentrations in the neuroinflammatory group compared with controls. (**b**) Activity concentrations in blood, lung, liver, spleen, and kidney, showing comparable values between experimental groups. (**c**) Brain-to-blood ratios indicating an increased ratio in the neuroinflammatory group. (**d**) Organ weights, showing no significant differences between groups. Statistically significant results were visually marked as ⋆ significant.

Ex vivo γ-counter analysis of blood and harvested organs calculated a radioactivity concentration of (7.49 ± 0.33) %IA/g in the blood of neuroinflammatory mice (Fig. 4b), consistent with levels observed via PET imaging. In the brain (Fig. 4a), radiotracer accumulation was significantly higher in the neuroinflammatory model ((0.28 ± 0.002) %IA/g) compared to control mice ((0.21 ± 0.04) %IA/g; *p* = 0.03), while blood levels in controls remained comparable at (6.70 ± 0.72) %IA/g. The brain-to-blood ratios 24 h *p.i.* (Fig. 4c) were (20.7 ± 1.6) % in the neuroinflammatory group and (15.0 ± 1.5) % in the control group (*p* < 0.01). Radiotracer uptake in peripheral organs, including the liver, spleen, kidneys, and lungs (Fig. 4b) was similar across both groups with no significant differences observed. Furthermore, organ weights recorded 24 h *p.i.* showed no significant variations between the models (Fig. 4d).

**Figure 5:**
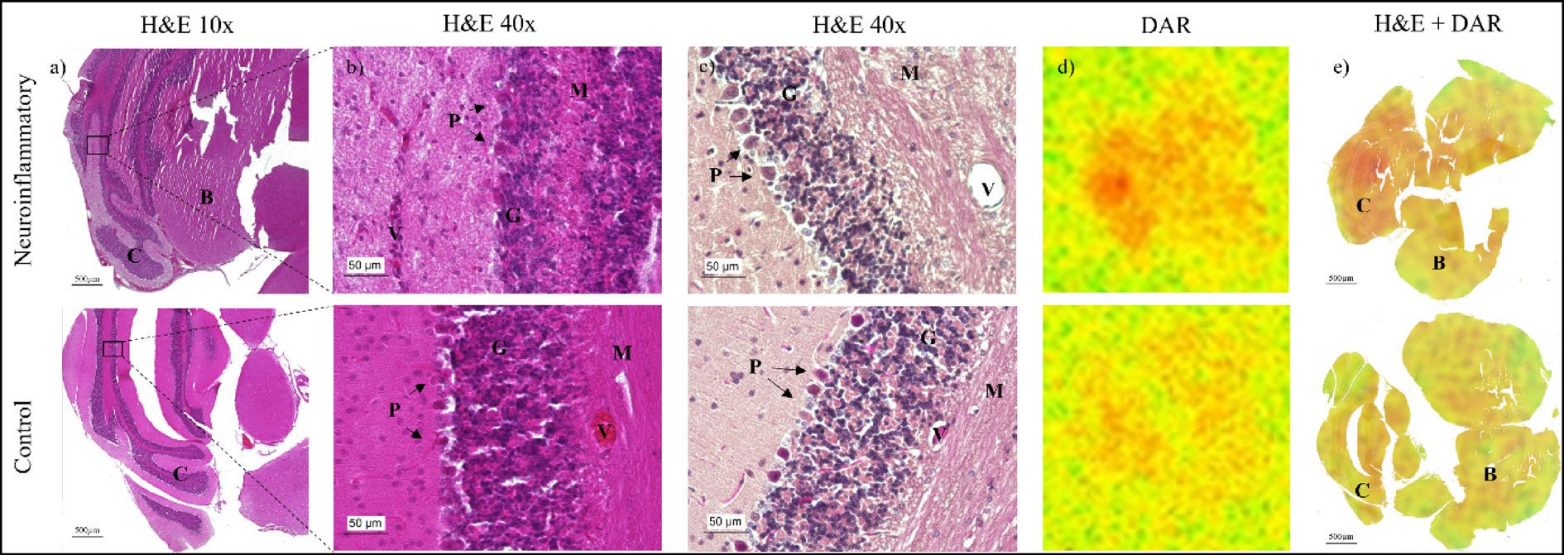
Histological and digital autoradiography (DAR) analysis of brain sections from control and neuroinflammatory model mice. Hematoxylin and eosin (H&E) staining showing (**a**) an overview of the cerebrum (B) and cerebellum (C), (**b**) a 40 × magnification of the cerebellar cortex (10 µm section thickness) and (**c**) a 40 × magnification of the subsequent adjacent section of the same region (4 µm section thickness). The cerebellar cortical layers are indicated, including blood vessel (V), the granule cell layer (G), molecular layer (M), and Purkinje cell layer (P; black arrows indicate Purkinje cells). No apparent histopathological differences were observed between the experimental groups. DAR analysis and co-registered H&E and DAR (**d**,**e**) revealed a stronger cerebellar signal in the neuroinflammatory group compared with controls. Scale bars: (a and e) 500 µm; (**b**,**c**) 50 µm.

Digital autoradiography (DAR) analysis co-registered with H&E-stained cerebral and cerebellar sections (Fig. 5d,e) suggested a more pronounced radiotracer signal in the cerebellum of neuroinflammatory mice compared with controls. Corresponding histological evaluation of the cerebellar parenchyma (Fig. 5a–c) confirmed that the cerebellar architecture was preserved in both neuroinflammatory and control groups. Specifically, the Purkinje and granule cell layers appeared morphologically normal, with no signs of cell loss or structural degradation observed in either cohort (Fig. 5b,c).

## Discussion

### Multimodal PET and MR imaging of neuroinflammatory and control mice

In this study, the overall in vivo biodistribution of [^89^Zr]Zr-DFO-HSA was successfully documented in both neuroinflammatory and control mice. The inflammatory degree of the NMRI transgenic mice (IKK2-CA^GFAP^ strain) has been previously validated, showing a robust induction of NF-κB-dependent pro-inflammatory cytokines such as TNF-α and IL-6^23^. The pharmacokinetics of the radiotracer were similar between the neuroinflammatory and control models. Both groups exhibited high initial intravascular activity followed by a continuous decline over the observation period. By 24 h *p.i.*, prominent signals were observed in abdominal organs, particularly the liver, consistent with the expected clearance pathways for this radiotracer^21,25^. These findings suggest that the presence of localized neuroinflammation does not significantly alter the systemic pharmacokinetic profile of the radiolabeled HSA.

### Impact of chronic neuroinflammation on the BBB integrity

Anatomical MRI revealed no evidence of anticipated neuroinflammatory effects on brain tissue, such as ventricular enlargement, reduced cerebral grey matter volume, or altered signal intensities^27,28^. These observations are consistent with the early phase of chronic neuroinflammation in this model, where significant morphological alterations are not typically expected. Previous studies have similarly indicated that macroscopic manifestations of neuroinflammation in this neuroinflammatory mouse model, such as structural cerebellar atrophy, emerge only at later stages^23^. Histological analyses corroborated these findings, as H&E staining confirmed the absence of observable cellular changes between the cohorts. The congruence between MRI and histology in both the current study and previous reports^24,29^ emphasizes the lack of structural damage at this early stage across all examined brain regions, especially the cerebellum. Interestingly, PET imaging and DAR analysis revealed distinct differences in cerebral and cerebellar radiotracer distribution between the two mouse models. Specifically, the neuroinflammatory mice exhibited a stronger radiotracer signal in the cerebellar region 24 h *p.i.*, highlighting this area as a primary site of increased radiotracer permeability.

To quantify this, brain-to-blood radioactivity ratios were employed as a robust surrogate marker for radiotracer extravasation. The progressive longitudinal increase in these ratios within the neuroinflammatory group indicates the enhanced accumulation of albumin within the brain parenchyma. Given the high molecular weight of albumin and its negligible physiological transport into the CNS^30^, these elevated ratios serve as a reliable indicator of macromolecular leakage.

At early time points (1 h* p.i*.), accurate quantification of intracerebral radiotracer concentrations was likely confounded by partial volume effects (PVE) resulting from high radioactivity within the small-caliber meningeal vessels. This intravascular signal leads to an inherent overestimation of parenchymal activity, particularly in regions adjacent to the vascular compartments^20,21^. However, by 24 h *p.i*., the observable clearance of the radiotracer from the systemic circulation reduced the impact of PVE, thereby improving the accuracy of the measurements. While in vivo PET imaging continued to slightly overestimate cerebral radioactivity compared to ex vivo γ-counter analysis, the observed differences between the cohorts remains distinct. Ultimately, these results indicate that [^89^Zr]Zr-DFO-HSA PET/MR imaging effectively identifies and localizes increased BBB permeability in the context of neuroinflammation.

The findings of this PET study align with prior research on the IKK2-CA^GFAP^ neuroinflammatory mouse model, which identified the cerebellum as the primary region susceptible to the effects of chronic neuroinflammation during advanced disease stages^34,23^. Despite the recognized potential for early therapeutic intervention, non-invasive diagnostic tools for early detection have remained elusive^23^. This study indicated that HSA-based PET/MR imaging can detect neuroinflammatory effects on blood–brain barrier permeability at nascent stages of disease progression. In this context, increased BBB permeability and macromolecular leakage may serve as robust surrogate markers of barrier dysfunction. This technique allows for the precise localization and quantification of altered radiotracer extravasation well before the emergence of overt morphological changes, emphasizing the sensitivity of [^89^Zr]Zr-DFO-HSA as a molecular imaging tool.

## Conclusion and limitations

This study provides preliminary evidence for the feasibility of [^89^Zr]Zr-DFO-HSA PET/MR imaging in detecting and visualizing altered BBB permeability during chronic neuroinflammation. Despite the limited cohort size, this non-invasive approach allowed for the localization of region-specific tracer accumulation—particularly within the cerebellum—prior to the onset of overt cerebellar symptoms. However, technical challenges in PET quantification were observed. High radioactivity within the meningeal vasculature resulted in partial volume effects that confounded the isolation of parenchymal signals, leading to an overestimation of intracerebral concentrations. These limitations, likely amplified by the small scale of the murine vasculature, would likely be less pronounced in a clinical setting due to the significantly larger diameter of human vessels. In conclusion, these exploratory findings suggest that [^89^Zr]Zr-DFO-HSA PET/MR is a promising diagnostic tool for the sensitive detection of macromolecular leakage. While further validation in larger cohorts is warranted, this approach holds potential for investigating barrier dysfunction within clinically relevant and complex pathological frameworks, such as neuroinflammatory disorders and traumatic brain injury.

## Supplementary Information

Below is the link to the electronic supplementary material.

Supplementary Material 1.

Supplementary Material 2.

## Data Availability

All data generated or analyzed during this study are included in this published article and its supplementary information files; additional data are available from the corresponding author upon reasonable request.
