# Supplementary material for: Detection of inflammation-related blood–brain barrier dysfunction using PET and MR imaging: a pilot study
Source: Sci Rep. 2026 Apr 10;16:12014. doi: 10.1038/s41598-026-47352-6 (PMC13069071; doi:10.1038/s41598-026-47352-6)
Supplement: Supplementary file 1 — Supplementary Material 1. [file 41598_2026_47352_MOESM1_ESM.docx]

**S.1. covalent attachment of Desferal (DFO) to HSA**

A 10 mM solution of p-SCN-DFO (Macrocyclics Inc., Plano, Texas, USA) was prepared in DMSO (Sigma-Aldrich, St. Louis, Missouri, USA). Subsequently, 13 mg of HSA (Sigma-Aldrich, St. Louis, Missouri, USA) was dissolved in 4 mL of phosphate-buffered saline (PBS, Biochrom, Berlin, Germany) and adjusted to pH 9 using 0.1 M Na_2_CO_3_. A 3- to 4-fold molar excess (80 µL of 10 mM solution) of p-SCN-DFO was added to the HSA solution. The mixture was incubated at 37°C for 24 *h*. The resulting HSA-DFO conjugate was purified using a 30 kDa centrifugal filter unit (Amicon Ultra 30 K, 2 mL, Merk Millipore, Carrigtohill, Ireland; Micro 220R Hettich Centrifuges, Tuttlingen, Germany). Protein concentration was determined using a Coomassie Protein Assay (Thermo Fisher Scientific, Waltham, MA, USA) according to the manufacturer's protocol, with absorbance read at 595 nm on a Multiskan™ GO Microplate Spectrophotometer (Thermo Fisher Scientific, Waltham, MA, USA). Radiometric isotope dilution assays revealed an average of 4.48 DFO per HSA protein were bound (Supplements S.1.1).

**S.1.1.1 Number of chelating agents Desferal per HSA**

The number of zirconium-89 accessible DFO chelating agents conjugated to the HSA was determined by modified radiometric isotope dilution assays following the protocol Zeglis B. M. et al [49] protocol. Basically, the complexation capacity of pure DFO with zirconium-89 was investigated in order to compare it with the complexation capacity of DFO-HSA. For this purpose, 3 mg DFO was dissolved in 1 ml DMSO and then a dilution series of DFO (5 nmol - 0.05 pmol) was prepared. These were incubated with an average activity of 1.79 ± 0.12 MBq [89Zr]ZrCl4 for 24 h at room temperature. At the end of the incubation period, the extent of complex formation from the added [^89^Zr]ZrCl4 to the DFO was determined by thin-layer chromatography in a phosphor imager (Fujifilm, Minato, Japan) (see 2.1.3).

At the same time, 15.3 µg DFO-HSA was dissolved in 0.9 % isotonic NaCl solution (B. Braun SE, Melsungen, Germany) and incubated with different activity levels of zirconium-89 (0.05 MBq - 3.8 MBq). The mixtures were incubated for 24 h at room temperature. Subsequently, the complexed zirconium-89 amount was also determined by thin-layer chromatography and graphically represented (see 2.1.3). Based on the linear equation of the graphs obtained, the complexing capacity of the DFO and the DFO-HSA was determined and thus the number of DFO per HSA protein was derived.

**S.1.1.2. Calculation of the chelator number per HSA protein**

The number of desferal (DFO) per molecule of human serum albumin (HSA) was determined using the radioisotope dilution assay.

The dose-dependent complexation capacity of DFO with 1.7 MBq of zirconium-89 was evaluated using a dilution series of DFO (Fig. 1 a). At a dose of 0.02 µg DFO (27 pmol), a complexation efficiency of 4% was observed, while doses exceeding 1.68 µg DFO (3 nmol) resulted in complexation efficiencies greater than 96%.

In contrast, the complexation capacity of 15.3 µg DFO-HSA was assessed using a dilution series of zirconium-89 radioactivity. At a zirconium-89 activity of 0.4 MBq, 15.3 µg DFO-HSA successfully complexed over 96% of the zirconium-89 isotopes. However, when the activity was increased to 1.7 MBq and 3.8 MBq, the complexation efficiencies decreased to 54% and 28%, respectively.

**Figure 7:** **The successful coupling of DFO to HSA.** 1.7 MBq zirconium-89 was incubated with an ascending dilution series of DFO (3 fmol - 3 nmol) for 24 h (a). The percentage of complexed zirconium atoms was plotted in each case. The complexation capacity of 15.3 µg DFO-HSA was plotted using an ascending dilution series of 89Zr (b). When 1.7 MBq zirconium-89 (6.84*1011 zirconium-89 atoms) was incubated with 15.3 µg DFO-HSA (1.39*1014 particles of HSA-DFO), the same amount of zirconium-89 atoms was complexed as with 0.58 µg DFO (6.24*1014 DFO molecules). Accordingly, an average of 4.48 DFO molecules were bound per HSA molecule.

These results indicate that 15.3 µg DFO-HSA (1.39 × 10^14^ particles), when incubated with 1.7 MBq zirconium-89 (6.84 × 10^11^ [^89^Zr]Zr isotopes), achieved comparable complexation values to 0.58 µg DFO (6.24 × 10^14^ DFO molecules). This suggests that, on average, 4.48 functional and zirconium-89 accessible DFO molecules were bound per HSA molecule.

**S.1.2. Hematoxylin and Eosin (H&E) staining and digital autoradiography (DAR).**

Brains were placed in cassettes (KABE Labortechnik, Germany), fixed overnight in 4% formaldehyde solution (Thermo Fisher Scientific, Waltham, MA, USA) and embedded in liquid paraffin. Sections were prepared using a rotary microtome (Leica JUNG RM2045, Wetzlar, Germany), producing 4 µm thick slices for H&E staining and 10 µm thick slices for DAR which were mounted on microscope slides (Merck KGaA, Darmstadt, Germany). DAR slides were placed on Europium imaging plates (Fujifilm, Tokyo, Japan), incubated for 30 days, then scanned with a fluorescence laser scanner.

Concerning H&E staining, the general protocol is outlined in accordance to Hilbrig et al. [12]. 4 µm thick tissue sections were deparaffinized using xylene and a descending alcohol series, stained with hematoxylin (Waldeck GmbH & Co. KG, Münster, Germany) for 10 min and 2% eosin solution (Waldeck GmbH & Co. KG, Münster, Germany) for two minutes in water. Stained sections were evaluated by BZ-X810 microscope (Keyence, Neu-Isenburg, Germany) at 4x and 10x magnification.
