# Supplementary material for: Detection of inflammation-related blood–brain barrier dysfunction using PET and MR imaging: a pilot study
Source: Sci Rep. 2026 Apr 10;16:12014. doi: 10.1038/s41598-026-47352-6 (PMC13069071; doi:10.1038/s41598-026-47352-6)
Supplement: Supplementary file 2 — Supplementary Material 2. [file 41598_2026_47352_MOESM2_ESM.docx]

*Table 1: Quantitative in vivo biodistribution analysis neuroinflammatory mouse model PET imaging 1 h [%IA/mL]*

| Time [h] | Brain | | | Blood | | | Whole body | | |
| --- | --- | --- | --- | --- | --- | --- | --- | --- | --- |
| 0,00 | 0,31 | 0,17 | 0,19 | 0,80 | 0,15 | 0,13 | 0,20 | 0,14 | 0,18 |
| 0,01 | 2,74 | 0,97 | 0,62 | 18,47 | 19,79 | 8,79 | 2,35 | 0,99 | 0,18 |
| 0,01 | 3,89 | 3,02 | 1,55 | 29,18 | 28,10 | 27,89 | 4,53 | 3,09 | 0,20 |
| 0,02 | 3,56 | 2,98 | 1,60 | 27,47 | 23,67 | 31,75 | 4,43 | 3,44 | 0,17 |
| 0,03 | 3,66 | 3,18 | 2,55 | 27,47 | 22,50 | 32,43 | 4,37 | 3,45 | 0,77 |
| 0,03 | 3,90 | 3,33 | 2,86 | 24,79 | 23,38 | 25,56 | 4,40 | 3,38 | 2,86 |
| 0,04 | 2,88 | 2,70 | 1,97 | 24,36 | 22,38 | 20,70 | 4,00 | 3,17 | 2,68 |
| 0,06 | 2,87 | 2,53 | 1,64 | 25,02 | 21,93 | 14,49 | 3,95 | 3,14 | 2,62 |
| 0,08 | 2,74 | 2,18 | 1,63 | 23,55 | 22,05 | 12,99 | 3,94 | 3,06 | 2,66 |
| 0,09 | 2,79 | 2,46 | 1,67 | 23,77 | 21,93 | 12,58 | 3,98 | 3,02 | 2,63 |
| 0,11 | 2,80 | 2,37 | 1,66 | 24,21 | 21,13 | 12,95 | 3,85 | 3,04 | 2,64 |
| 0,13 | 2,84 | 2,45 | 1,69 | 22,68 | 21,09 | 13,66 | 3,83 | 3,04 | 2,57 |
| 0,14 | 2,60 | 2,48 | 1,81 | 22,16 | 21,48 | 12,64 | 3,85 | 3,02 | 2,60 |
| 0,19 | 2,18 | 2,35 | 1,59 | 21,67 | 21,37 | 14,05 | 3,77 | 3,04 | 2,50 |
| 0,28 | 2,06 | 2,43 | 1,50 | 20,71 | 20,78 | 14,17 | 3,74 | 3,08 | 2,48 |
| 0,36 | 2,07 | 2,36 | 1,49 | 20,37 | 19,91 | 13,76 | 3,71 | 3,03 | 2,47 |
| 0,44 | 1,98 | 2,26 | 1,47 | 21,07 | 20,16 | 14,03 | 3,70 | 3,04 | 2,46 |
| 0,53 | 1,95 | 2,47 | 1,41 | 20,33 | 19,88 | 13,25 | 3,68 | 2,95 | 2,47 |
| 0,61 | 2,03 | 2,33 | 1,43 | 20,24 | 19,75 | 12,86 | 3,65 | 2,92 | 2,45 |
| 0,69 | 2,02 | 2,44 | 1,39 | 19,84 | 18,87 | 11,97 | 3,64 | 2,90 | 2,44 |
| 0,78 | 2,01 | 2,37 | 1,45 | 20,04 | 19,54 | 11,57 | 3,61 | 2,92 | 2,44 |
| 0,86 | 1,90 | 2,39 | 1,57 | 18,94 | 18,20 | 11,51 | 3,60 | 2,91 | 2,43 |
| 0,94 | 1,92 | 2,24 | 1,50 | 19,68 | 18,59 | 11,47 | 3,58 | 2,85 | 2,41 |

*Table 2: Quantitative in vivo biodistribution analysis control mouse model PET imaging 1 h [%IA/mL]*

| Time [h] | Brain | | | | Blood | | | | Whole body | | | |  |
| --- | --- | --- | --- | --- | --- | --- | --- | --- | --- | --- | --- | --- | --- |
| 0,00 | 0,65 | 0,24 | 0,16 | 0,37 | 1,73 | 0,16 | 0,09 | 0,22 | 0,32 | 0,22 | 0,18 | 0,30 | |
| 0,01 | 3,12 | 0,39 | 0,21 | 0,29 | 23,17 | 3,17 | 0,16 | 0,83 | 2,23 | 0,68 | 0,19 | 0,62 | |
| 0,01 | 3,54 | 2,64 | 0,25 | 0,63 | 22,70 | 21,44 | 0,10 | 2,38 | 3,23 | 4,16 | 0,19 | 1,58 | |
| 0,02 | 3,89 | 2,41 | 0,22 | 0,94 | 22,25 | 18,34 | 0,95 | 2,97 | 3,48 | 4,59 | 0,36 | 2,33 | |
| 0,03 | 3,56 | 2,21 | 0,85 | 1,01 | 22,19 | 18,51 | 4,34 | 3,26 | 3,52 | 4,73 | 1,30 | 2,45 | |
| 0,03 | 3,57 | 2,56 | 1,29 | 1,03 | 21,78 | 17,62 | 7,11 | 4,21 | 3,64 | 4,59 | 1,88 | 2,65 | |
| 0,04 | 2,47 | 1,78 | 1,08 | 0,77 | 19,11 | 16,90 | 9,73 | 3,39 | 2,86 | 4,12 | 2,30 | 2,27 | |
| 0,06 | 2,40 | 1,71 | 1,21 | 0,79 | 19,32 | 16,94 | 11,50 | 3,49 | 2,88 | 4,12 | 2,67 | 2,36 | |
| 0,08 | 2,65 | 1,72 | 1,32 | 1,24 | 18,11 | 16,32 | 10,81 | 7,91 | 2,83 | 4,11 | 2,73 | 2,39 | |
| 0,09 | 2,16 | 1,62 | 1,38 | 1,54 | 18,10 | 16,58 | 11,12 | 11,67 | 2,88 | 4,11 | 2,63 | 2,44 | |
| 0,11 | 2,91 | 1,76 | 1,46 | 1,62 | 17,98 | 16,87 | 10,87 | 11,61 | 2,90 | 4,06 | 2,63 | 2,42 | |
| 0,13 | 2,42 | 1,69 | 1,36 | 1,34 | 19,09 | 16,08 | 10,68 | 10,90 | 2,85 | 4,07 | 2,63 | 2,40 | |
| 0,14 | 2,83 | 1,75 | 1,34 | 1,44 | 19,20 | 15,89 | 10,46 | 11,12 | 2,85 | 4,11 | 2,58 | 2,36 | |
| 0,19 | 1,89 | 1,58 | 1,12 | 1,10 | 17,93 | 16,06 | 10,52 | 10,87 | 2,67 | 3,98 | 2,48 | 2,16 | |
| 0,28 | 1,90 | 1,59 | 1,08 | 1,11 | 17,12 | 15,50 | 10,60 | 10,46 | 2,64 | 3,93 | 2,46 | 2,15 | |
| 0,36 | 1,76 | 1,59 | 1,13 | 1,05 | 16,25 | 15,58 | 10,34 | 10,57 | 2,61 | 3,89 | 2,44 | 2,12 | |
| 0,44 | 1,76 | 1,65 | 1,04 | 1,09 | 16,56 | 15,31 | 10,05 | 10,68 | 2,60 | 3,84 | 2,42 | 2,12 | |
| 0,53 | 1,65 | 1,63 | 1,07 | 1,07 | 17,07 | 15,09 | 9,78 | 10,53 | 2,62 | 3,83 | 2,44 | 2,10 | |
| 0,61 | 1,62 | 1,56 | 1,05 | 1,07 | 16,20 | 15,11 | 9,76 | 10,62 | 2,60 | 3,82 | 2,38 | 2,11 | |
| 0,69 | 1,68 | 1,66 | 0,96 | 1,07 | 16,15 | 15,14 | 10,43 | 10,62 | 2,60 | 3,79 | 2,40 | 2,11 | |
| 0,78 | 1,56 | 1,62 | 1,06 | 1,07 | 16,70 | 15,18 | 9,83 | 10,62 | 2,62 | 3,78 | 2,38 | 2,11 | |
| 0,86 | 1,59 | 1,50 | 1,03 | 1,07 | 16,77 | 14,92 | 9,88 | 10,62 | 2,64 | 3,78 | 2,37 | 2,11 | |
| 0,94 | 1,53 | 1,65 | 1,03 | 1,07 | 16,46 | 15,06 | 9,88 | 10,62 | 2,62 | 3,81 | 2,76 | 2,11 | |

*Table 3: Quantitative in vivo biodistribution analysis neuroinflammatory mouse model PET imaging 24 h [%IA/mL]*

| Time [h] | Brain | | | Blood | | | Whole body | | |
| --- | --- | --- | --- | --- | --- | --- | --- | --- | --- |
| 24,04 | 1,35 | 1,09 | 0,97 | 5,88 | 7,75 | 5,11 | 2,88 | 2,16 | 2,79 |
| 24,13 | 1,13 | 0,93 | 0,98 | 6,16 | 7,45 | 5,00 | 2,88 | 2,16 | 2,80 |
| 24,21 | 1,36 | 1,00 | 0,99 | 5,44 | 7,30 | 4,78 | 2,87 | 2,14 | 2,77 |
| 24,29 | 1,04 | 0,98 | 0,98 | 6,14 | 7,03 | 4,66 | 2,86 | 2,12 | 2,78 |
| 24,38 | 1,10 | 1,06 | 0,96 | 6,01 | 7,17 | 4,59 | 2,87 | 2,11 | 2,78 |
| 24,46 | 1,22 | 0,98 | 0,98 | 5,83 | 6,91 | 4,44 | 2,86 | 2,10 | 2,75 |
| 24,54 |  | 0,97 | 1,00 |  | 6,96 | 4,78 | 2,86 | 2,12 | 2,80 |
| 24,63 |  | 0,98 | 0,97 |  | 6,78 | 4,50 | 2,86 | 2,08 | 2,77 |
| 24,71 |  | 0,95 | 0,99 |  | 6,78 | 4,46 | 2,85 | 2,08 | 2,77 |
| 24,79 |  | 0,85 | 0,96 |  | 7,10 | 4,23 | 2,87 | 2,06 | 2,78 |
| 24,88 |  | 1,01 | 0,94 |  | 6,93 | 4,47 | 2,87 | 2,07 | 2,77 |

*Table 4: Quantitative in vivo biodistribution analysis control mouse model PET imaging 24 h [%IA/mL]*

| Time [h] | Brain | | | | Blood | | | | Whole body | | | |
| --- | --- | --- | --- | --- | --- | --- | --- | --- | --- | --- | --- | --- |
| 24,04 | 0,91 | 0,84 | 0,84 | 0,81 | 5,73 | 6,54 | 4,13 | 4,87 | 2,60 | 3,05 | 1,70 | 2,09 |
| 24,13 | 0,95 | 0,88 | 0,83 | 0,77 | 5,64 | 6,51 | 4,02 | 4,84 | 2,60 | 3,04 | 1,71 | 2,08 |
| 24,21 | 0,87 | 0,93 | 0,81 | 0,76 | 5,25 | 6,48 | 4,19 | 4,89 | 2,56 | 3,06 | 1,72 | 2,09 |
| 24,29 | 0,86 | 0,95 | 0,79 | 0,73 | 5,74 | 6,29 | 4,05 | 4,75 | 2,59 | 3,04 | 1,73 | 2,08 |
| 24,38 | 0,81 | 0,86 | 0,81 | 0,79 | 5,63 | 6,48 | 3,97 | 4,74 | 2,57 | 3,04 | 1,72 | 2,10 |
| 24,46 | 0,86 | 0,86 | 0,79 | 0,79 | 5,69 | 6,32 | 4,01 | 4,66 | 2,56 | 3,06 | 1,72 | 2,08 |
| 24,54 |  | 0,86 | 0,76 | 0,78 |  | 6,38 | 3,93 | 4,56 | 2,60 | 3,04 | 1,71 | 2,09 |
| 24,63 |  | 0,79 | 0,80 | 0,78 |  | 6,18 | 3,91 | 4,50 | 2,56 | 3,05 | 1,72 | 2,07 |
| 24,71 |  | 0,80 | 0,75 | 0,82 |  | 6,30 | 4,04 | 4,64 | 2,59 | 3,03 | 1,72 | 2,08 |
| 24,79 |  | 0,91 | 0,85 | 0,82 |  | 6,54 | 3,91 | 4,68 | 2,56 | 3,05 | 1,70 | 2,06 |
| 24,88 |  | 0,99 | 0,81 | 0,77 |  | 6,23 | 3,91 | 4,66 | 2,58 | 3,02 | 1,72 | 2,07 |

*Table 5: Quantitative ex vivo biodistribution analysis neuroinflammatory mouse model [%IA/g]*

| Tissue | NI + | | |
| --- | --- | --- | --- |
| Blood sample | 7,39 | 7,93 | 7,14 |
| Heart | 3,10 | 4,14 | 2,26 |
| Brain | 0,28 | 0,28 | 0,28 |
| Lung | 3,80 | 4,12 | 2,93 |
| Liver1 | 3,59 | 5,55 | 4,48 |
| Liver 2 | 4,92 | 5,13 | 4,21 |
| Spleen | 4,26 | 5,09 | 3,01 |
| Kidney | 3,03 | 1,75 | 2,31 |
| Small intestine | 1,72 | 2,87 | 1,63 |
| Large intestine | 1,98 | 2,70 | 1,52 |
| Muscle sample | 0,78 | 1,26 | 0,81 |
| Bone sample | 1,84 | 1,47 | 0,76 |
| Tail | 1,85 | 2,79 | 2,86 |

*Table 6: Quantitative ex vivo biodistribution analysis control mouse model [%IA/g]*

| Tissue | NI - | | | |
| --- | --- | --- | --- | --- |
| Blood sample | 7,38 | 7,27 | 6,57 | 5,57 |
| Heart | 2,84 | 3,40 | 2,11 | 1,47 |
| Brain | 0,20 | 0,27 | 0,18 | 0,17 |
| Lung | 5,49 | 4,40 | 3,24 | 2,14 |
| Liver1 | 4,32 | 5,95 | 4,30 | 3,84 |
| Liver 2 | 4,15 | 5,83 | 4,11 | 3,77 |
| Spleem | 4,06 | 4,56 | 3,11 | 2,51 |
| Kidney | 2,36 | 2,66 | 2,39 | 2,11 |
| Small intestine | 2,19 | 1,87 | 1,35 | 0,80 |
| Large intestine | 1,87 | 1,61 | 1,71 | 0,88 |
| Muscle sample | 0,89 | 0,93 | 0,71 | 0,58 |
| Bone sample | 1,49 | 1,25 | 0,91 | 0,67 |
| Tail | 1,53 | 1,72 | 3,26 | 0,90 |

*Table 7: Ex vivo organ weight neuroinflammatory mouse model [g]*

| Tissue | NI + | | |
| --- | --- | --- | --- |
| Heart | 3,10 | 4,14 | 2,26 |
| Brain | 0,28 | 0,28 | 0,28 |
| Lung | 3,80 | 4,12 | 2,93 |
| Liver | 3,59 | 5,55 | 4,48 |
| Spleen | 4,26 | 5,09 | 3,01 |
| Kidney | 3,03 | 1,75 | 2,31 |

*Table 8: Ex vivo organ weight control mouse model [g]*

| Tissue | NI - | | | |
| --- | --- | --- | --- | --- |
| Heart | 2,84 | 3,40 | 2,11 | 1,47 |
| Brain | 0,20 | 0,27 | 0,18 | 0,17 |
| Lung | 5,49 | 4,40 | 3,24 | 2,14 |
| Liver | 4,32 | 5,95 | 4,30 | 3,84 |
| Spleen | 4,06 | 4,56 | 3,11 | 2,51 |
| Kidney | 2,36 | 2,66 | 2,39 | 2,11 |


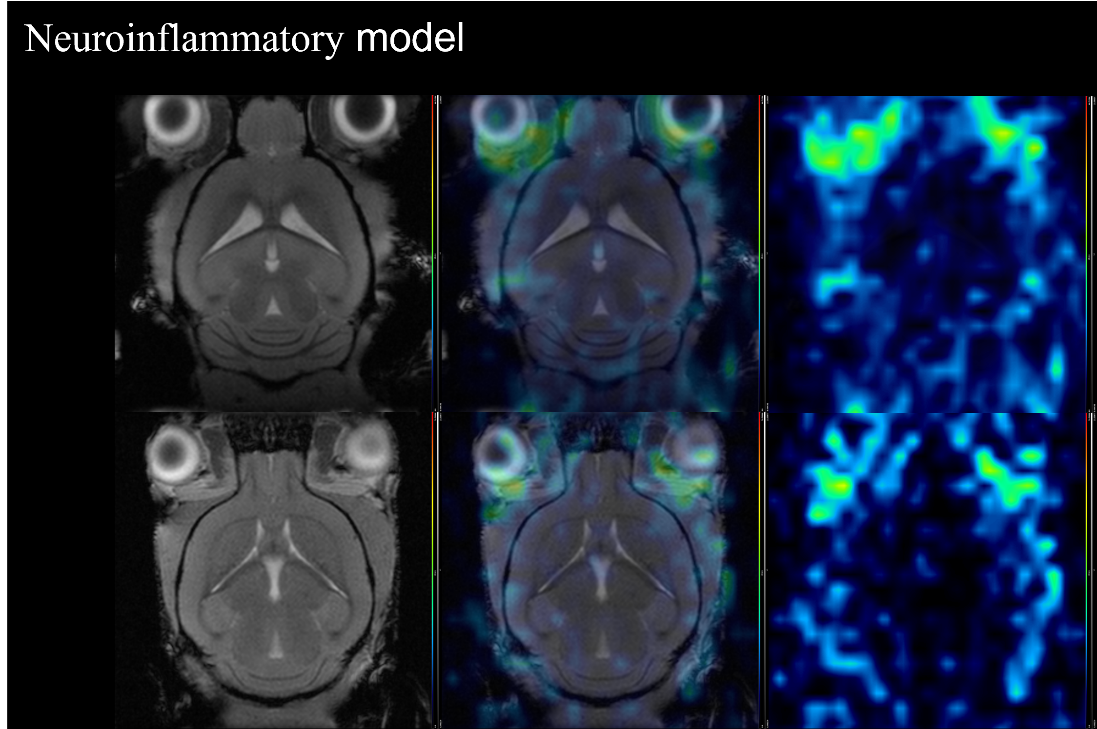


Figure 1: cranial PET and MR images of neuroinflammatory mice.


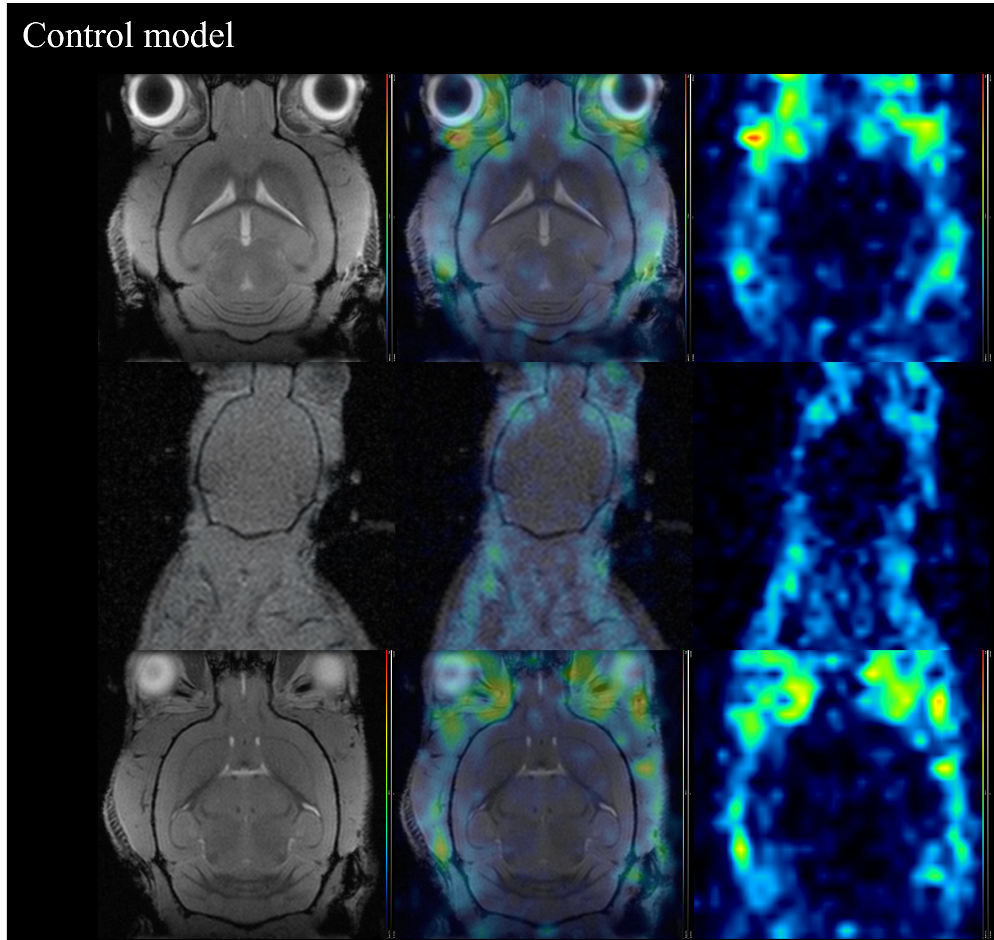


Figure 2: cranial PET and MR images control mice.


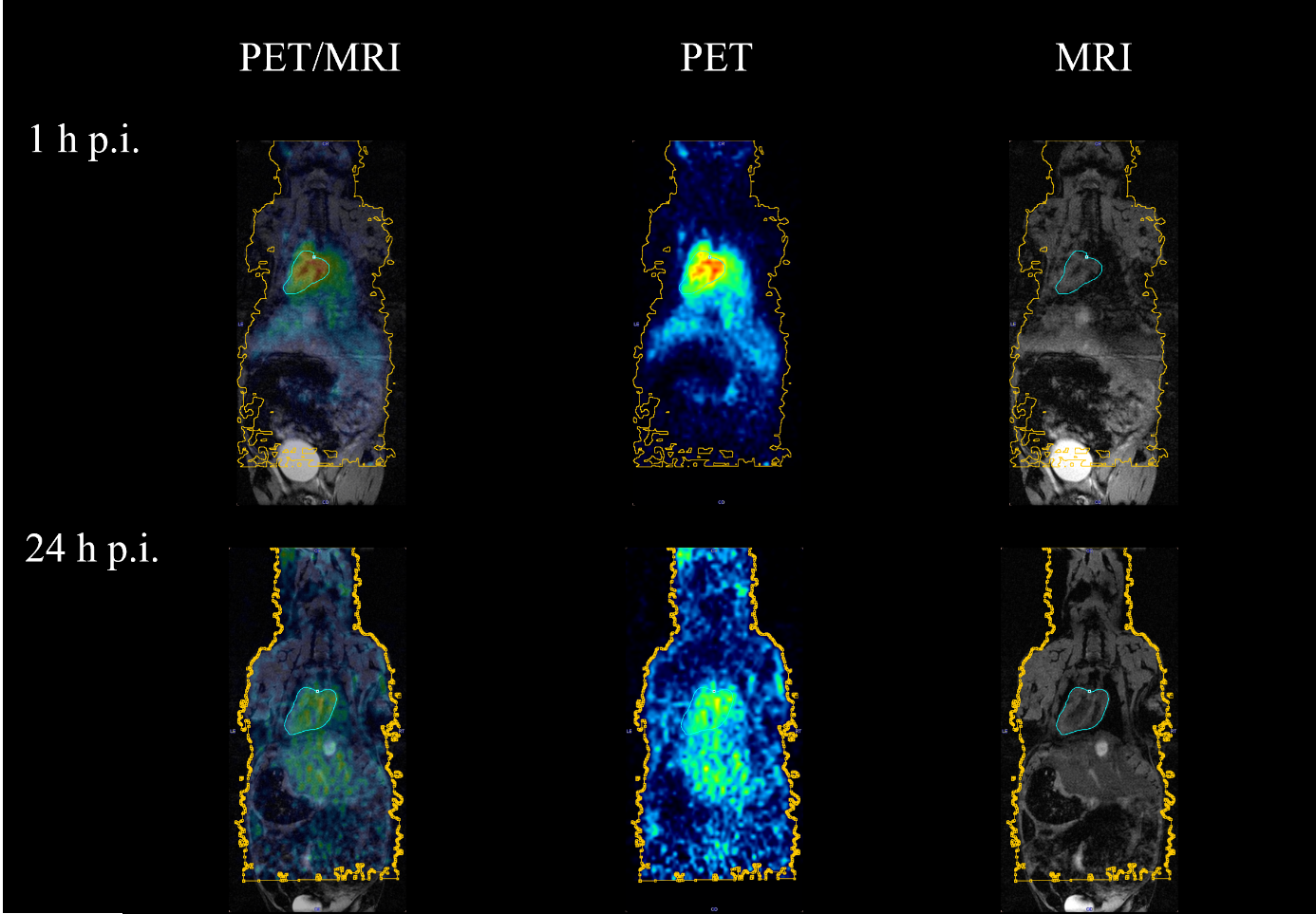


Figure 3: VOI analysis of bloodpool and whole body mouse based on PET and MRI images.


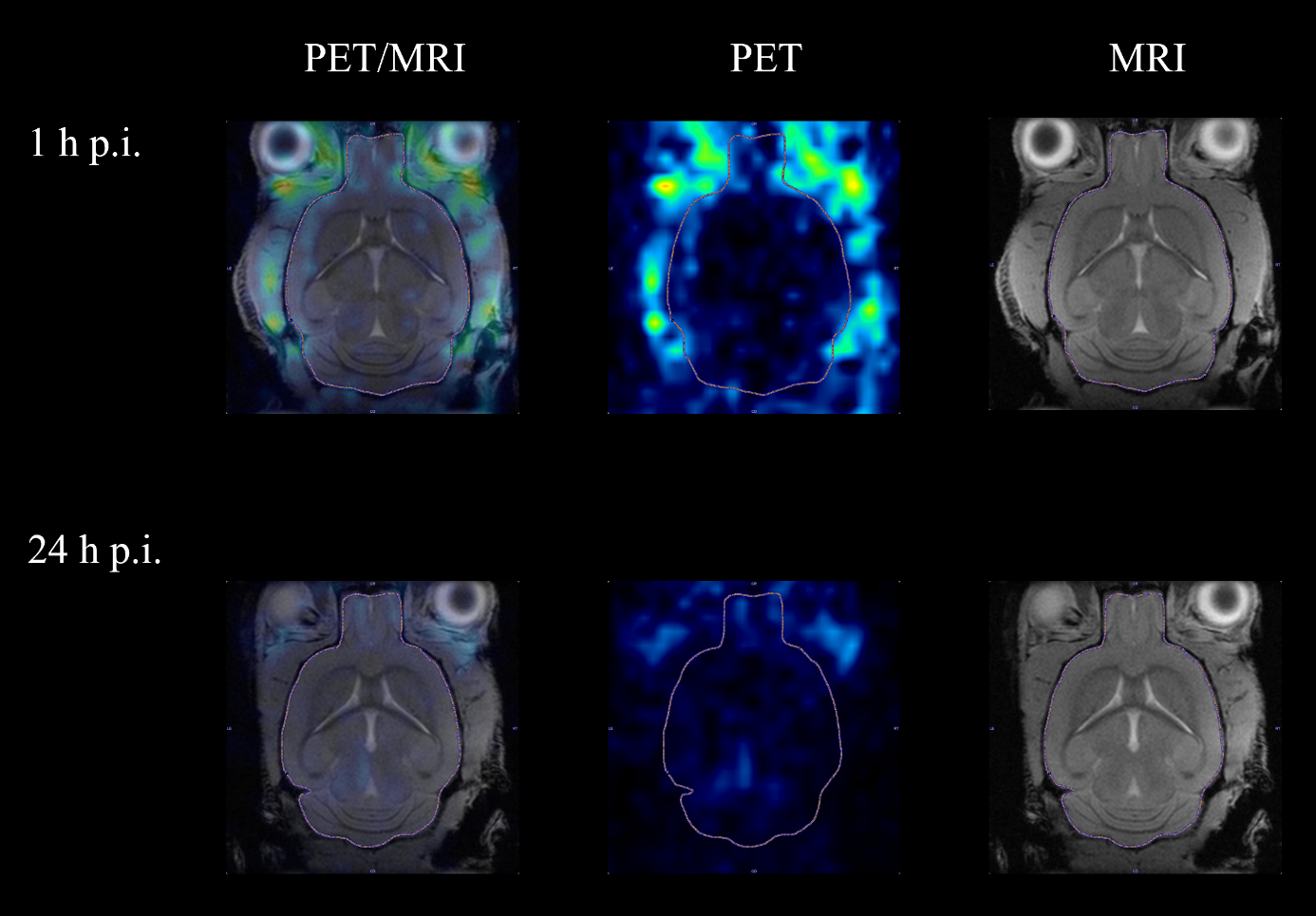


Figure 4: VOI analysis of brain based on PET and MRI images

Figure 5: Individual time activity curves of whole body, blood and brain over 24 h.
